# Upfront dexrazoxane for the reduction of anthracycline-induced cardiotoxicity in adults with preexisting cardiomyopathy and cancer: a consecutive case series

**DOI:** 10.1186/s40959-019-0036-7

**Published:** 2019-01-29

**Authors:** Sarju Ganatra, Anju Nohria, Sachin Shah, John D. Groarke, Ajay Sharma, David Venesy, Richard Patten, Krishna Gunturu, Corrine Zarwan, Tomas G. Neilan, Ana Barac, Salim S. Hayek, Sourbha Dani, Shantanu Solanki, Syed Saad Mahmood, Steven E. Lipshultz

**Affiliations:** 1 Cardio-Oncology Program, Lahey Hospital and Medical Center, Burlington, MA USA; 2 Department of Cardiovascular Medicine, Lahey Hospital and Medical Center, Burlington, MA USA; 3 Cardio-Oncology Program, Department of Cardiovascular Medicine, Brigham and Women’s Hospital, Boston, MA USA; 4 Department of Hematology Oncology, Lahey Hospital and Medical Center, Burlington, MA USA; 5 Cancer Survivorship Program, Lahey Hospital and Medical Center, Burlington, MA USA; 6 Cardio-Oncology Program, Division of Cardiology, Massachusetts General Hospital, Boston, MA USA; 7 Cardio-Oncology Program, Division of Cardiology, Medstar Washington Hospital Center, Washington, DC USA; 8 Division of Cardiovascular Medicine, University of Michigan, Ann Arbor, MI USA; 9 Division of Cardiovascular Medicine, Eastern Maine Medical Center, Bangor, ME USA; 10 Department of Medicine, Westchester Medical Center, Valhalla, NY USA; 11 Division of Cardiovascular Medicine, New-York Presbyterian Hospital/Weill Cornell Medical Center, New York City, NY USA; 12 Department of Pediatrics, University at Buffalo Jacobs School of Medicine and Biomedical Sciences, Oishei Children’s Hospital, Roswell Park Comprehensive Cancer Center, Buffalo, NY USA

**Keywords:** Anthracycline, Cardiotoxicity, Dexrazoxane, Cardiomyopathy, Cardioprotection

## Abstract

**Background:**

Cardiotoxicity associated with anthracycline-based chemotherapies has limited their use in patients with preexisting cardiomyopathy or heart failure. Dexrazoxane protects against the cardiotoxic effects of anthracyclines, but in the USA and some European countries, its use had been restricted to adults with advanced breast cancer receiving a cumulative doxorubicin (an anthracycline) dose > 300 mg/m^2^. We evaluated the off-label use of dexrazoxane as a cardioprotectant in adult patients with preexisting cardiomyopathy, undergoing anthracycline chemotherapy.

**Methods:**

Between July 2015 and June 2017, five consecutive patients, with preexisting, asymptomatic, systolic left ventricular (LV) dysfunction who required anthracycline-based chemotherapy, were concomitantly treated with off-label dexrazoxane, administered 30 min before each anthracycline dose, regardless of cancer type or stage. Demographic, cardiovascular, and cancer-related outcomes were compared to those of three consecutive patients with asymptomatic cardiomyopathy treated earlier at the same hospital without dexrazoxane.

**Results:**

Mean age of the five dexrazoxane-treated patients and three patients treated without dexrazoxane was 70.6 and 72.6 years, respectively. All five dexrazoxane-treated patients successfully completed their planned chemotherapy (doxorubicin, 280 to 300 mg/m^2^). With dexrazoxane therapy, changes in LV systolic function were minimal with mean left ventricular ejection fraction (LVEF) decreasing from 39% at baseline to 34% after chemotherapy. None of the dexrazoxane-treated patients experienced symptomatic heart failure or elevated biomarkers (cardiac troponin I or brain natriuretic peptide). Of the three patients treated without dexrazoxane, two received doxorubicin (mean dose, 210 mg/m^2^), and one received daunorubicin (540 mg/m^2^). Anthracycline therapy resulted in a marked reduction in LVEF from 42.5% at baseline to 18%. All three developed symptomatic heart failure requiring hospitalization and intravenous diuretic therapy. Two of them died from cardiogenic shock and multi-organ failure.

**Conclusion:**

The concomitant administration of dexrazoxane in patients with preexisting cardiomyopathy permitted successful delivery of anthracycline-based chemotherapy without cardiac decompensation. Larger prospective trials are warranted to examine the use of dexrazoxane as a cardioprotectant in patients with preexisting cardiomyopathy who require anthracyclines.

## Introduction

Since their introduction in the early 1950s, anthracyclines have been widely used for treating a variety of solid and hematologic cancers in both adults and children [1]. However, cardiotoxicity has limited their use in patients with preexisting cardiomyopathy or symptomatic heart failure [2]. In the early 1970s, Eugene Herman discovered that dexrazoxane reduced anthracycline-induced cardiotoxicity [3]. In 1995, the US Food and Drug Administration (FDA) approved dexrazoxane for cardioprotection in women receiving fluorouracil, doxorubicin, and cyclophosphamide (FDC) chemotherapy for breast cancer and who required a cumulative doxorubicin dose > 350 mg/m^2^ [4]. Similarly, in 2011 the European Medicines Agency (EMA) decided that the use of dexrazoxane should be restricted to adult breast cancer patients requiring a cumulative dose of doxorubicin > 300 mg/m^2^ or a cumulative dose of epirubicin > 540 mg/m^2^ [5]. This limitation comes from debatable concerns that dexrazoxane might reduce anti-tumor response rates and increase the risk of secondary hematologic malignancies [6–9].

We sought to determine whether dexrazoxane would be cardioprotective in patients without the current indications for its use. Here, we present a consecutive series of patients from a single institution with preexisting cardiomyopathy, who were treated with anthracyclines, and compare their cardiovascular and cancer-related outcomes to those of similar patients who subsequently received concomitant off-label dexrazoxane with anthracycline-based chemotherapy.

## Methods

In the two years preceding the initiation of the Lahey cardio-oncology program (July 2013 to June 2015), we noted that several patients with either preexisting cardiomyopathy, or those who experienced anthracycline-induced cardiomyopathy, developed worsening LV systolic function and major adverse cardiovascular events including heart failure, cardiogenic shock, arrhythmias, and cardiovascular death, despite the use of maximally tolerated, guideline-directed heart failure therapy.

**Fig. 1:**
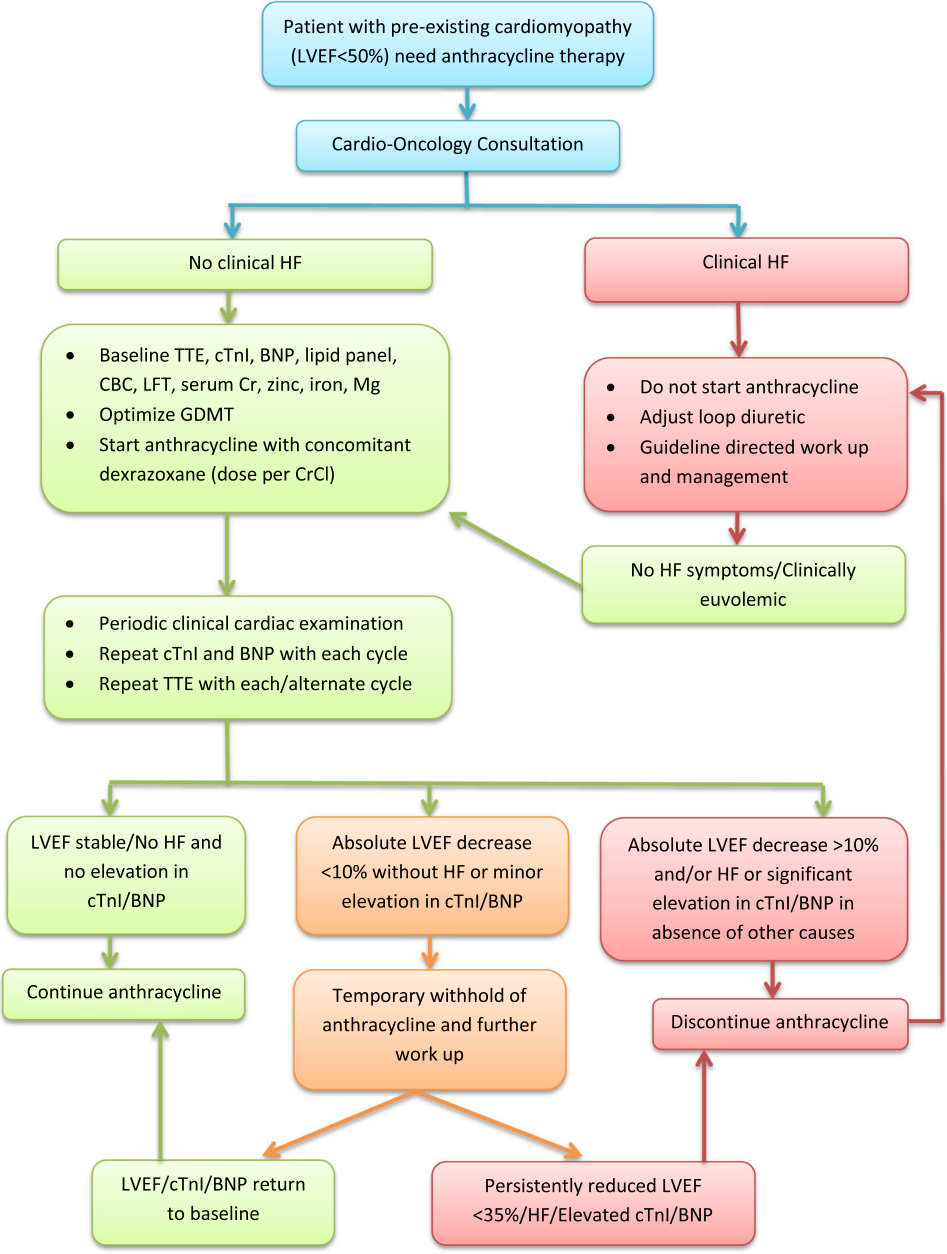
Clinical protocol for patients with existing cardiomyopathy requiring anthracycline therapy

After evaluating the evidence on the use of dexrazoxane for cardioprotection against anthracycline-induced cardiotoxicity, we developed a clinical practice algorithm (Fig. 1) based on consensus among internal and external experts in cardio-oncology, hematology-oncology, and heart failure to use off-label concomitant dexrazoxane, in addition to guideline-directed heart failure therapy and close cardiac monitoring, in subsequent patients with preexisting cardiomyopathy who required anthracycline-based chemotherapy. Since this was a consensus-based clinical practice guideline, and not an experimental research study, institutional review board approval was not required.

### Patient selection

Beginning in July 2015, five consecutive patients with preexisting asymptomatic LV systolic dysfunction, who required anthracycline-based chemotherapy, were treated with dexrazoxane as a cardioprotectant as per our consensus protocol. Three consecutive patients with preexisting asymptomatic LV systolic dysfunction who received anthracycline based chemotherapy between July 2013 and June 2015 were identified for comparison.

### Cardioprotection protocol

All five patients received off-label dexrazoxane in a 10:1 ratio of dexrazoxane to doxorubicin, or at a reduced dose based on their creatinine clearance, 30 min before each anthracycline dose [4] throughout the therapy, regardless of cancer type or stage. All patients also received maximally tolerated, guideline-directed heart failure therapy [10]. Complete blood count, liver function, kidney function, and zinc, iron, and magnesium concentrations were assessed at baseline and after each chemotherapy cycle. Serum cardiac biomarkers including cardiac troponin I (cTnI) and brain natriuretic peptide (BNP) were measured before and within 48 h after each dose of anthracyclines [11–13], as well as 6 months after completion of chemotherapy. Echocardiograms were obtained at baseline, before every subsequent or alternate cycle of chemotherapy (as per the primary cardiology or cardio-oncology provider), immediately after the completion of anthracycline-based chemotherapy, and 6 months later [12].

### Patient follow-up

All patients receiving dexrazoxane were followed in our cardio-oncology clinic, either before each subsequent or alternate cycle of anthracycline chemotherapy, as deemed appropriate by their providers. All patients were thoroughly examined for signs or symptoms of heart failure. We also collected data on cardiovascular and cancer-related outcomes (asymptomatic LV systolic dysfunction, heart failure, arrhythmias, cardiogenic shock, elevated serum cardiac biomarker concentrations defined as cTnI > 0.03 ng/mL and/or BNP > 250 pg/mL, tumor response, secondary malignancy, or death from any cause) during follow-up. Patients were followed until death or loss to follow-up.

## Results

### Patients treated without Dexrazoxane

Between July 2013 and June 2015, we had treated three consecutive male patients treated with anthracycline but without dexrazoxane (patients #1, #2, and #3; mean age, 72.6 years) at the Lahey Hospital and Medical Center. Patient #1 had preexisting asymptomatic non-ischemic cardiomyopathy (LVEF 40%), patient #2 had preexisting asymptomatic ischemic cardiomyopathy (LVEF 45%), and patient #3 had anthracycline-induced cardiomyopathy that developed after receiving a cumulative daunorubicin dose of 270 mg/m^2^ for acute myeloid leukemia (LVEF 45%) (Table 1). Each patient in this group had received maximally tolerated doses of guideline-directed heart failure therapy. Patients #1 and #2 received doxorubicin (cumulative doses of 300 mg/m^2^ and 120 mg/m^2^, respectively), and patient #3 received daunorubicin (cumulative dose 540 mg/m^2^).

**Table 1:** Clinical and Treatment Characteristics of Eight Patients with Preexisting Cardiomyopathy Undergoing Anthracycline Chemotherapy for Any Cancer, With or Without the Cardioprotectant, Dexrazoxane

Patient #. sex; age, years	Cancer, Stage	Chemotherapy regimen; anthracycline, cumulative dose, mg/m^2^	Cardiac history	Before the start of anthracycline chemotherapy			LVEF, %, by time since anthracycline therapy			
				ACEi	Beta Blocker	Aldosterone Antagonist	Pre	During	Post	6 months post
Without dexrazoxane (controls)										
1. M; 87	PTCL, IV	CHOP Doxorubicin, 300	NICM	Yes	Yes	Yes	45	–	10	–
2. M; 66	DLBCL, IV	R-EPOCH Doxorubicin, 120	Ischemic Cardiomyopathy	Yes	Yes	No	40	15	–	–
3. M; 65	AML, M4	Cytarabine + Daunorubicin Daunorubicin, 540	Chemotherapy- related cardiomyopathy	Yes	No	Yes	55	45	30	30
With dexrazoxane										
4. F; 67^a^	HL, IIIB	ABVD Doxorubicin, 300	NICM	Yes	Yes	No	35	35	35	25
5. F; 75	DLBCL, IIISE	CHOP Doxorubicin, 300	NICM	Yes	Yes	Yes	35	35	35	30
6. F 70	Ovarian, IV	Doxorubicin, 280	NICM	No	Yes	No	45	40	40	40
7. M; 73	NHL, IV	ABVD Doxorubicin, 300	Ischemic cardiomyopathy	Yes	Yes	Yes	40	40	40	40
8. F; 68	Breast, IV	Doxorubicin Doxorubicin, 300	NICM	Yes	Yes	No	40	40	40	35

*LVEF* left ventricular ejection fraction, *ACEi* angiotensin-converting enzyme inhibitors, *DEX* dexrazoxane, *PTCL* peripheral T-cell lymphoma, *CHOP* cyclophosphamide, doxorubicin, vincristine, and prednisone, *NICM* non-ischemic cardiomyopathy, *DLBCL* diffuse large B-cell lymphoma, *M4* acute myelomonocytic leukemia, *R-EPOCH* rituximab, etoposide phosphate, prednisone, vincristine sulfate (Oncovin), cyclophosphamide, and doxorubicin hydrochloride (hydroxydaunorubicin), *AML* acute myeloid leukemia, *ABVD* Adriamycin (doxorubicin), Bleomycin, Vinblastine, Dacarbazine, *HL* Hodgkin lymphoma

^a^Patient had a defibrillator before chemotherapy

All three patients in this group experienced marked reductions in LV systolic function (the mean LVEF decreased from 42.5% at baseline to 18% after chemotherapy), and developed symptomatic heart failure requiring hospitalization and intravenous diuretic therapy, either during or within the first six months after completion of chemotherapy (Table 2). Patients #1 and #2 developed fatal cardiogenic shock and multi-organ failure. Patient #2 had advanced, diffuse, large B-cell lymphoma and received only two cycles of anthracycline-based chemotherapy before experiencing heart failure, cardiogenic shock, and death. Patient #3 had acute myeloid leukemia and cardiomyopathy that developed during chemotherapy with daunorubicin (LVEF 45%). He continued therapy and received an additional 270 mg/m^2^ of daunorubicin with further worsening of his cardiomyopathy (LVEF 30%) that persisted, despite treatment with neurohormonal antagonists.

**Table 2:** Outcomes of Eight Patients with Preexisting Cardiomyopathy Undergoing Anthracycline Chemotherapy for Any Cancer, With or Without The Cardioprotectant, Dexrazoxane

Patient #. Sex; age, years	Elevated biomarker concentrations^a^		Clinical HF	New arrhythmia	Clinical outcome	Chemotherapy cycles received, n	Cancer outcomes
	Troponin	BNP					
Without dexrazoxane							
1. M; 87	Not available	Not available	Yes	NSVT	Cardiogenic shock, HF, death	6	Died
2. M; 66	Not available	Not available	Yes	No	Cardiogenic shock, HF, death	3	Died
3. M; 65	Not available	Not available	Yes	No	Required ICD	2	Alive at 12 months after Allo SCT
With dexrazoxane							
4. F; 67	No	No	No	NSVT	Alive at 30 months	6	Complete remission
5. F; 75	No	No	No	No	Alive at 18 months, Needed BiV ICD implantation	6	Complete remission
6. F 70	No	No	No	No	Alive at 12 months	6	Partial response
7. M; 73	No	No	No	No	Alive at 15 months	6	Complete remission
8. F; 68	No	No	No	NSVT	Alive at 12 months	6	Complete response

*NSVT* Non-sustained ventricular tachycardia, *HF* heart failure, *ICD* implantable cardiodefibrillator, *Allo SCT* allogeneic stem cell transplantation, *BiV ICD* bi-ventricular implantable cardiodefibrillator

^a^Threshold values for elevation were > 0.03 ng/mL for cTnI and > 250 pg/mL for BNP

### Patients treated with Dexrazoxane

The dexrazoxane protocol was implemented in the next five consecutive eligible patients (patients #4 through #8; mean age, 70.6 years), one man and four women, four with non-ischemic cardiomyopathy and one with ischemic cardiomyopathy. The mean pre-chemotherapy LVEF for these five dexrazoxane-treated patients was 39% (range, 35 to 45%) (Table 1). Patient #4 had an implantable cardioverter-defibrillator for primary prevention. All five patients received doxorubicin (280 to 300 mg/m^2^).

The median follow-up period was 13.5 months (range, 12–30 months). Two patients treated without dexrazoxane died and none were lost to follow-up.

The mean post-chemotherapy LVEF in this group was 34% and all five completed planned chemotherapy without any major adverse cardiovascular events (Table 2). Patient #5, with a preexisting left bundle branch block and non-ischemic cardiomyopathy (baseline LVEF 35%), required cardiac resynchronization therapy with a bi-ventricular implantable cardioverter-defibrillator after chemotherapy, given that her LVEF persistently remained < 35%. None of the patients experienced symptomatic heart failure requiring intravenous diuresis or hospitalization, and no secondary malignancies were detected ≥12 months after completion of chemotherapy.

Serum concentrations of cTnI and BNP were not elevated in any of the five patients. Patients #4 and #8 had sporadic episodes of non-sustained ventricular tachycardia, but no sustained arrhythmias were noted (Table 2).

Three of the five patients in this group experienced significant neutropenia (absolute neutrophil count < 500/μL), and two patients experienced neutropenic fever requiring treatment with antibiotics. None of the patients developed any detectable end-organ damage as a result. No clinically important abnormalities were noted in any other laboratory parameters including serum liver function, kidney function, and zinc, iron, and magnesium concentrations.

## Discussion

The patients in our case series had preexisting cardiomyopathy that placed them at increased risk for cardiotoxicity from anthracycline-based chemotherapy. Off-label use of concomitant dexrazoxane as a cardioprotectant allowed successful administration of planned anthracycline-based chemotherapy, without symptomatic cardiac decompensation. In contrast, similar patients who received anthracyclines without cardioprotection, experienced a significant decline in their LVEF, coupled with adverse cardiovascular outcomes including death. It is also important to note that in our case series, dexrazoxane was not associated with either a reduced anti-tumor response or the development of secondary malignancies during the median follow-up period of 13.5 months. Our data, although limited, support the use of concomitant dexrazoxane as a cardioprotectant in patients with preexisting cardiomyopathy who require anthracycline therapy.

### The overlap of Cancer and cardiomyopathy

With an aging population, and with an increase in the number of patients with cardiovascular disease (CVD) and cancer survivors, greater overlap between CVD and cancer is expected [14–17]. The risk of anthracycline-induced cardiotoxicity is increased in the elderly, patients with cardiovascular comorbidities, and in patients receiving additional cardiotoxic chemotherapies or thoracic irradiation [18]. Our case series includes patients with preexisting LV systolic dysfunction who are often excluded from receiving anthracycline-based chemotherapy due to the risk of worsening cardiomyopathy. This often results in inferior cancer outcomes, or in cases where they receive anthracyclines, increased cardiovascular morbidity and mortality. In this case series, we report our observations on the successful use of dexrazoxane as a cardioprotectant in this patient population.

### Anthracycline-related cardiotoxicity

Anthracyclines have been widely used to treat a variety of solid and hematological cancers in children and adults since the 1950s [1]. Cardiac complications were reported a few years later [19], but anthracyclines remain one of the most commonly used classes of chemotherapeutic agents, given their oncologic efficacy [1].

Anthracycline-related cardiotoxicity ranges from subclinical cardiomyopathy to florid heart failure. Cardiotoxicity may occur within the first week of anthracycline treatment or even decades later [20]; however, most cases occur within the first year after treatment [21]. The frequency and characteristics of both clinical and subclinical cardiotoxicity may vary between children and adults [22]. In adult breast cancer patients, the incidence of cardiotoxicity is approximately 9% [21]. The actual incidence of late cardiotoxicity in long-term survivors of childhood cancer is difficult to ascertain since it increases with longer survival, but up to one third of childhood survivors have some evidence of cardiac dysfunction, including overt heart failure [23].

Doxorubicin is not the only anthracycline associated with cardiotoxicity; epirubicin and daunorubicin, other commonly used anthracyclines to treat several cancers, have also caused similar cardiotoxicity, especially at high doses [24–26]. In this case series as well, one of the patients in the anthracycline-treated group who did not receive dexrazoxane (patient #5) experienced daunorubicin-related cardiotoxicity resulting in cardiomyopathy and heart failure.

Anthracyclines are believed to cause cardiotoxicity by multiple mechanisms; including both the generation of oxygen free radicals and by inhibition of topoisomerase 2B. Substantial experimental data support the involvement of iron in anthracycline-induced cardiotoxicity, including evidence suggesting the importance of the HFE gene in regulating cardiac iron accumulation after exposure to anthracyclines, irrespective of a patient’s systemic iron load [27].

### Preventing cardiomyopathy

#### Dexrazoxane: Mechanism of action

Dexrazoxane is an iron chelator that binds free iron or removes iron from the doxorubicin-iron complex, thereby preventing oxygen free radical formation. This property may explain how dexrazoxane reduces some of the cardiotoxic effects of anthracyclines [28]. However, other iron chelators, such as deferasirox, or ICRF-161, are not cardioprotective [29, 30]. Dexrazoxane also changes the configuration of topoisomerase II [31] thus preventing anthracyclines from binding to the topoisomerase II complex [32]. This may be an additional mechanism for the cardioprotective effects of dexrazoxane.

#### Dexrazoxane: Facts and fiction (Table 3)

The current approved use of dexrazoxane in adults is based on two multicenter, randomized controlled trials (088001 and 088006) involving more than 500 women with advanced breast cancer [6]. In these studies, women were randomized to receive doxorubicin, with or without dexrazoxane (10:1 dexrazoxane:doxorubicin). In study 088001, 15% of women who received dexrazoxane and 31% of those who received placebo experienced a cardiac event (HR 2.63, 95%CI 1.61 to 4.27). Similarly, in study 088006, 14% of those treated with dexrazoxane experienced a cardiovascular event compared to 31% treated with placebo (HR 2.0, 95% CI 1.01 to 3.96). Cardiac events were defined as a decline in LVEF from baseline ≥10% and below the institution’s lower limit of normal (LLN), a decline in LVEF of ≥20% from baseline, or the development of heart failure (with two or more of the following: cardiomegaly established by radiography, basilar rales, S3 gallop, or paroxysmal nocturnal dyspnea, orthopnea, or significant dyspnea on exertion) [6]. The early signal of substantial cardioprotection in these studies, led to the use of open-label dexrazoxane in patients on placebo who had received a cumulative doxorubicin dose of 300 mg/m^2^. These patients experienced a marked reduction in cardiovascular events, despite the late introduction of dexrazoxane (HR 3.5, 95% CI 2.2 to 5.7) [6, 7].

**Table 3:** Safety and Efficacy of Dexrazoxane in Clinical Studies

First Author and Year of Publication	Population	Anthracycline Agent	Number of Subjects	Median Age (years)	Baseline CV Risk Factors	Dexrazoxane Administration Timing	Cardiotoxicity in Dexrazoxane Arm	Cardiotoxicity in Control Arm	Reduced anti-tumor efficacy	Incidence of Secondary Malignancy
Swain SM et al. 1997 [6] Prospective RCT, Dex vs. Placebo	Adult, Advanced breast cancer	Doxorubicin	534	58 (study 088001) 56 (study 088006)	No difference between two groups	From the beginning of therapy	15% (study 088001) 14% (study 088006)	31% (study 088001) 31% (study 088006)	Lower response rate (14% difference) but no effect on overall time to progression or survival (study 088001)	Not evaluated
Swain SM et al. 1997 [7] Prospective RCT, Dex vs. Placebo	Adult, Advanced breast cancer	Doxorubicin	201	57	No difference between two groups	After a cumulative doxorubicin dose of 300 mg/m^2^	3%	22%	No effect	Not evaluated
Rabinovich A et al. 2012 [34] Retrospective, comparing cancer outcomes in Dex vs. No-Dex	Adult, NHL	Doxorubicin	193	70.35	Dex group had more CV risk factors	61.7% of the patients started dexrazoxane in the first or second chemotherapy cycle	Not evaluated	Not evaluated	No effect	Not evaluated
Limat S et al. 2014 [35] Retrospective, comparing two groups based on time of treatment (1994–2000 and 2001–2005)	Adult, NHL	Doxorubicin	180	58	No difference between two groups	From the beginning of therapy (Note: Only 45% patients in 2001–05 group received Dex)	17%	1.5%	Not evaluated	Not evaluated
Vachhani P et al. 2017 [36] Retrospective, consecutive patients with AML treated with Dex, baseline LVEF 40–50%	Adult, AML	Daunorubicin or Mitoxantrone	6	61.5	No control group	50% (3/6) had Dex from the beginning of therapy. The rest 3 had Dex after median of 340 mg/m^2^ anthracycline	16.67% (1/6 patients)	No control arm	Not evaluated	Not evaluated
Lopez M et al. 1998 [38] Prospective RCT, Dex vs. No Dex	Adult, Advanced breast cancer or soft tissue sarcoma	Epirubicin	Breast cancer – 95 Sarcoma - 34	Breast cancer −58 Sarcoma - 51	Not mentioned in the study. Dose of epirubicin was higher in Dex arm	From the beginning of therapy	4.1%	20.8%	No effect	Not evaluated
Schuler MK et al. 2016 [39] Retrospective, Dex given for one of the following reasons: 1. Re-challenge, 2. Reaching the cumulative anthracycline dose and 3. Preexisting heart failure	Adult, Sarcoma	Doxorubicin	32	54	No control group	27/32 patients received Dex after a median dose of 450 mg/m2 of doxorubicin 5/32 patients received Dex from the beginning of therapy (Given baseline elevated cardiac risk – CHF, CAD or arrhythmia)	7%	No control arm	Not evaluated	Not evaluated
Lipshultz SE et al. 2004 [3] Prospective RCT, Dex vs. No Dex	Children, Previously untreated high-risk ALL	Doxorubicin	206	7.5	Not mentioned in the study but median cumulative dose of doxorubicin was same in both groups	From the beginning of therapy	21% (elevated cTnT) 10% (Extremely elevated cTnT level)	50% (elevated cTnT 32% (Extremely elevated cTnT level)	No effect	Not evaluated
Chow EJ at al. 2015 [40] Long term follow-up (median 12.6 years) of 3 prospective RCT patients to evaluate effect of Dex on long-term survival	Children, (T-cell acute lymphoblastic leukemia/ lymphoma, intermediate/ high-risk Hodgkin lymphoma, and low-risk Hodgkin lymphoma)	Doxorubicin	1008	12.6	Not mentioned in the study but median cumulative dose of doxorubicin was same in both groups in each trial	From the beginning of therapy	Not reported in the study but no difference in CV mortality	Not reported in the study but no difference in CV mortality	No effect	No effect
Asselin BL et al. 2016 [41] Prospective RCT, Dex vs. No Dex	Children, T-ALL or L-NHL	Doxorubicin	537	9.8	Not mentioned in the study but median cumulative dose of doxorubicin was same in both groups	From the beginning of therapy	Acute cardiotoxicity: 1 patient Elevated cTnT: 2.4% z scores for LV fractional shortening (3 years): −0.05 (normal)	Acute cardiotoxicity: 4 patient Elevated cTnT: 8.8% z scores for LV fractional shortening (3 years): − 0.77 (abnormal)	No effect	No effect
Tebbi CK et al. 2007 [8] Prospective RCT, Dex vs. No Dex	Children, Hodgkin’s disease treated with ABVE or dose-intensified ABVE-PC followed by low-dose radiation	Doxorubicin	478	12.9	Not reported	From the beginning of therapy	Not reported	Not reported	No effect	8/10 patients with secondary malignancy in Dex arm. Standardized incidence rate was 41.86 with Dex versus 10.08 without Dex (*P* = 0.0231)
Shaikh F et al. 2016 [9] Meta-analysis of 17 studies (5 RCT and 12 NRSs to evaluate effects of Dex for cardioprotection and secondary malignancy	Children, Variety of hematological and solid malignancies	Multiple agents (predominantly doxorubicin)	4639	Variable among studies (< 18)	Not reported	All but one NRS administered Dex from the beginning of anthracycline therapy	Dex reduced clinical or subclinical cardiotoxicity among RCTs (RR = 0.29, *P* = .003, NNT = 41) and NRSs (RR = 0.43, *P* < .001, NNT = 7)	No control arm	No effect	**RCT:** Dex: 2.7% No Dex: 1.1% (RR = 2.37, *P* = .06). Two RCTs that used concurrent etoposide reported an increased risk of AML, while one that used cranial radiation reported an increased risk of brain tumors. **NRSs:** No effect
Kim H et al. 2018 [42] Retrospective, Dex vs. No Dex	Children, Variety of hematological and solid malignancies	Multiple agents (predominantly doxorubicin)	1453	6	Not reported but anthracycline dose was higher in Dex arm (210 mg/m^2^ in Dex arm vs. 150 mg/m^2^ in No Dex arm, *p* < 0.01)	From the beginning of therapy	Cardiac event-free survival rate of patients with more than 400 mg/m^2^ of anthracyclines was significantly higher in Dex group	No control arm	Not evaluated	No effect
Getz KD et al. 2018 [43] Prospective, Comparing various treatments in children with AML, Dex given as per descrition of treating physician	Children, AML	Daunorubicin	1014 (only 96 had Dex)	Not reported in abstract	Not reported	From the beginning of therapy	Less declines in EF (∆EF: 0 to −4.0) Early LVSD – 6.3%	More significant decline in EF (∆EF: 0 to −6.4; *p* < 0.05) Early LVSD – 19.2% (*p* = 0.005)	Dex arm had non-significantly higher 3-year OS (71.9% vs 63.0%, *p* = 0.093) and EFS (54.4% vs 44.2%, *p* = 0.070)	Not evaluated

*ABVE* Adriamycin (doxorubicin), bleomycin, vincristine, etoposide, *ABVE-PC* Adriamycin (doxorubicin), bleomycin, vincristine, etoposide, prednisone, and cyclophosphamide, *AML* acute myeloid leukemia, *ALL* acute lymphoblastic leukemia, *cTn* cardiac troponin, *CV* cardiovascular, *Dex* dexrazoxane, *NHL* non-Hodgkin’s lymphoma, *LVEF* left ventricular ejection fraction, *LVSD* left ventricular systolic dyscuntion, *NRS* non-randomized study, *RCT* randomized control trial

In trial 088001, the dexrazoxane group had a lower antitumor response rate (complete remission and partial remission) when compared to the placebo group (48% vs. 63%, *P* = 0.007), although the overall time-to-progression and overall survival were not affected. Response rates were not significantly different in trial 088006. However, despite these disparate results, this led to a concern that dexrazoxane may reduce the anti-tumor efficacy of anthracyclines. Furthermore, in studies of children receiving dexrazoxane, there was an initial concern of an increased incidence of secondary malignancies, such as acute myeloid leukemia and myelodysplastic syndrome. Further follow-up of those same study patients has subsequently found that the concern for reduced oncologic efficacy was not correct [8]. Because the maximum benefit of dexrazoxane was seen in patients receiving a cumulative doxorubicin dose ≥300 mg/m^2^, and out of concern for reduced anti-tumor efficacy as well as increased risk of secondary malignancy, the FDA and EMA restricted its use to adults with advanced breast cancer receiving higher cumulative doses of doxorubicin [4–8]. The most recent American Society of Clinical Oncology guidelines also recommend limiting the use of dexrazoxane to patients receiving a cumulative doxorubicin dose > 250 mg/m^2^ [33].

Although dexrazoxane is not approved as a cardioprotectant in adults with hematologic malignancies, it has nevertheless been used in these patients. A retrospective analysis of adults with non-Hodgkin lymphoma treated with anthracycline-based combination chemotherapy revealed that dexrazoxane had similar cardioprotective effects [34, 35]. Dexrazoxane has also been shown to be cardioprotective in elderly patients with acute myeloid leukemia or small cell lung cancer [36, 37]. Similarly, dexrazoxane provided marked cardioprotection against epirubicin-related cardiotoxicity in patients with breast cancer or sarcoma with no evidence of reduced antitumor activity [38, 39].

Dexrazoxane has also been successfully used in children with various solid and hematologic cancers. The Dana-Farber Cancer Institute’s childhood acute lymphoblastic leukemia consortium protocols from 1995 to 2001 found that dexrazoxane reduced or prevented doxorubicin-induced cardiac injury in children with high-risk acute lymphoblastic leukemia, as indicated by lower serum cardiac troponin T concentrations and preserved LV systolic function and structure without reducing oncologic efficacy [3]. This has been found in multiple additional pediatric studies, including those from the Children’s Oncology Group [40, 41].

A single study is responsible for the unfounded fear that dexrazoxane reduces the oncologic efficacy of anthracyclines in adults, especially when used from the beginning of anthracycline therapy [6]. The results of this study have not been confirmed in other randomized trials and retrospective studies in children and adults with various cancers [40, 41].

As previously mentioned, secondary malignancies, such as acute myeloid leukemia and myelodysplastic syndrome, have been reported in studies of children receiving dexrazoxane [8]. However, this increased risk of secondary malignancies was only observed in two randomized controlled trials that administered etoposide with doxorubicin and dexrazoxane; in other randomized trials that did not include etoposide, the risk of acute myeloid leukemia or other secondary malignancies was not higher in the dexrazoxane arm [9]. Longer follow-up of the same study patients with the initial concern about secondary malignancies did not confirm this initial concern [40]. Similarly, a single randomized trial that administered cranial radiation to all patients (with either T-cell acute lymphoblastic leukemia or non-Hodgkin lymphoma) reported an increased rate of secondary brain tumors in the dexrazoxane arm. No brain tumors developed in any of the 717 dexrazoxane-treated patients enrolled in the other studies [9]. Overall, the concern for a higher incidence of secondary malignancies with dexrazoxane use is unwarranted. In retrospective analyses of long-term survivors of both childhood and adult cancers, dexrazoxane was not associated with an increase in secondary malignancies when used with doxorubicin chemotherapy [9, 41, 42]. Long-term follow-up (median follow-up, 12.6 years) of Children’s Oncology Group trials has established that dexrazoxane neither reduces long-term overall survival nor increases deaths from the original cancer [40]. In fact, dexrazoxane use in pediatric acute myeloid leukemia (AML) patients has shown better 3-year overall survival and event free survival [43].

Based on the efficacy of dexrazoxane as a cardioprotective agent in patients with a wide variety of cancers and with evolving safety data demonstrating that it does not impair chemotherapeutic efficacy and is not associated with an increased risk of secondary malignancies, in 2015 the European marketing authorization holder for Cardioxane (dexrazoxane; Clinigen Healthcare Ltd., Staffordshire, UK) submitted an application to all relevant national regulatory authorities to widen the indication from “advanced and/or metastatic adult breast cancer patients” to “cancer patients” and remove the contraindication in children and adolescents. The proposal to widen the indication was declined. However, after a careful review of dexrazoxane’s risk-benefit profile, the contraindication was lifted for children and adolescents requiring high doses (≥300 mg/m^2^) of anthracyclines [44].

Baseline LVEF is a significant predictor of anthracycline-induced cardiotoxicity [21]. Furthermore, while anthracycline-induced cardiotoxicity is dose-dependent, there is no threshold dose below which cardiotoxicity is not observed [11]. Thus, patients with preexisting cardiomyopathy are more susceptible to developing cardiotoxicity, even at lower anthracycline doses, and are often excluded from receiving anthracyclines altogether [18, 45]. Dexrazoxane could be particularly useful in these patients, but its use is limited by the current FDA and EMA label [4]. In this case series, we demonstrate that the off-label use of concomitant dexrazoxane in patients with preexisting, asymptomatic LV dysfunction, allows successful administration of anthracycline-based chemotherapy without adverse cardiovascular outcomes.

### Future perspective

Immunotherapies, including checkpoint inhibitors and adoptive cellular therapy, as well as targeted therapy, are now approved for several cancers and will likely change the landscape of oncology in coming years [46–48]. However, many patients will still need anthracycline chemotherapy to be cured. Cardiotoxicity is a well-known side effect of anthracyclines. Patients with preexisting cardiomyopathy are at the highest risk for anthracycline-related cardiotoxicity and are often denied lifesaving or sustaining anthracycline-based chemotherapy, even when there is no other alternative [18, 45]. Cardioprotection by dexrazoxane may offer a modality by which high-risk patients can receive anthracyclines without significantly increased cardiovascular morbidity and mortality [44].

Given that anthracycline-related cardiotoxicity can occur at low doses in patients with preexisting cardiomyopathy, our case series supports the use of dexrazoxane in this high-risk population from the beginning of anthracycline therapy, rather than after a cumulative dose of 300 mg/m^2^ is reached. Furthermore, we would advocate expanding its use to all cancers and not limiting it to patients with advanced breast cancer receiving doxorubicin. In patients with normal LV systolic function, the decision to administer dexrazoxane from the beginning of anthracycline therapy versus after receiving a cumulative dose of 300 mg/m^2^ may be debatable, but using dexrazoxane from the initiation of anthracycline therapy should be considered, regardless of the type of cancer [49].

Although dexrazoxane is cardioprotective, its protection is not complete and these patients should be monitored closely for signs of clinical and even subclinical cardiotoxicity. Although there is no question that signs of clinically significant cardiotoxicity may lead to interruption or reduction of anthracycline therapy, there is no consensus among our authors, or many in this field, that withholding or altering anthracycline therapy based solely on changes in subclinical cardiovascular status (changes in LVEF or serum cardiac biomarkers) will lead to a higher quality of life for these patients.

### Strengths and limitations of the study

This is a pragmatic study under real-world conditions. Although bias is possible in any case series, by including consecutive patients who met inclusion and exclusion criteria and comparing the outcomes with a historical group of anthracycline-treated patients who did not receive dexrazoxane, we attempted to minimize bias. Our objectives were clear, the outcomes were clinically relevant, and the data were prospectively collected. Finally, there was no loss to follow-up in either group. However, we recognize the limitations of findings based on only a few patients from a single center. Data collection in the patients treated earlier without dexrazoxane was limited by its retrospective nature.

## Conclusion

Our results, albeit limited, support the use of concomitant dexrazoxane for cardioprotection in patients with preexisting cardiomyopathy from the beginning of anthracycline therapy, regardless of cancer type or stage. Larger prospective trials are warranted to examine the expanded use of dexrazoxane in high-risk patients, including those with preexisting cardiomyopathy; cancers other than those affecting the breast; and without a minimum cumulative anthracycline dose.

## Abbreviations

BNP: Brain natriuretic peptide
cTnI: Cardiac troponin I
CVD: Cardiovascular disease
EMA: European Medical Agency
FDA: Food and Drug Administration
LV: Left ventricular
LVEF: Left ventricular ejection fraction
